# Platelet inhibitory effects of the Phase 3 anticancer and normal tissue cytoprotective agent, RRx‐001

**DOI:** 10.1111/jcmm.13791

**Published:** 2018-07-16

**Authors:** Bryan Oronsky, Neil Oronsky, Pedro Cabrales

**Affiliations:** ^1^ EpicentRx Inc La Jolla California; ^2^ CFLS Data Mountain View California; ^3^ Department of Bioengineering University of California San Diego La Jolla California

**Keywords:** cancer, haemoglobin, nitric oxide, red blood cells, RRx‐001

## Abstract

The platelet inhibitory effects of the Phase 3 anticancer agent and nitric oxide (NO) donor, RRx‐001, (1‐bromoacetyl‐3,3‐dinitroazetidine) were examined ex vivo and compared with the diazeniumdiolate NO donor, diethylenetriamine NONOate (DETA‐NONOate), which spontaneously releases nitric oxide in aqueous solution. In the absence of red blood cells and in a dose‐dependent manner, DETA‐NONOate strongly inhibited platelet aggregation induced by several stimuli (ADP, epinephrine and collagen) whereas RRx‐001 only slightly inhibited platelet aggregation under the same conditions in a dose‐dependent manner; these antiaggregant effects were blocked when both DETA‐NONOate and RRx‐001 were co‐incubated with carboxy‐PTIO (CPTIO 0.01‐100 micromol), a widely accepted NO scavenger. However, in the presence of red blood cells from healthy human donors, RRx‐001, which binds covalently to haemoglobin (Hb) and catalyses the production of NO from endogenous nitrite, more strongly inhibited the aggregation of platelets than DETA‐NONOate in a dose‐dependent manner likely because haemoglobin avidly scavenges nitric oxide and reduces its half‐life; the RRx‐001‐mediated platelet inhibitory effect was increased in the presence of nitrite. The results of this study suggest that RRx‐001‐bound Hb (within RBCs) plays an important role in the bioconversion of ${\text{NO}}_{2}^{-}$ to NO ^.^, which makes RRx‐001 a more physiologically relevant inhibitor of platelet aggregation than other nitric oxide donors, whose effects are attenuated in the presence of red blood cells. Therefore, RRx‐001‐mediated platelet inhibition is a potentially useful therapeutic property, especially in hypercoagulable cancer patients that are at an increased risk of thrombotic complications.

## 1 INTRODUCTION

It is well known that haemoglobin (Hb) in circulating red blood cells (RBCs) acts as a high‐affinity depot or storage pool for nitric oxide (NO)^1^ and that, cell‐free Hb, an even more avid scavenger of NO, contributes to the pathology of haemolytic anaemias like sickle cell disease, malaria and transfusion of older stored blood.^2^ Less well known is that nitrite enzymatically reacts with deoxyhaemoglobin to generate nitric oxide in the RBC, which serves as an “erythrocrine,” that is endocrine carrier and exporter of NO^.^ in the circulation under low oxygen conditions.^3^

Platelets are disc‐shaped cell fragments, 1/14th the volume of erythrocytes, that change shape and aggregate at wound sites to initiate clotting and stop bleeding in response to physical and chemical stimuli such as collagen,^4^ adenosine diphosphate (ADP), epinephrine, thromboxane A2 (TxA2) and thrombin. In addition to sealing vascular breaches, platelets are also implicated in the pathogenesis of hypertension, hypercholesterolaemia, cigarette smoking,^5^ diabetes and cancer^6^ and their activation predisposes to thrombotic vascular complications, leading to ischaemia and the development of coronary, cerebrovascular and peripheral artery disease. 7 Excessive activation, therefore, is tightly regulated, mostly through the action of inhibitory factors such as NO, released continually by eNOS (endothelial nitric oxide synthase) in the endothelium (except under hypoxic conditions when its activity is impaired), that limits both platelet adhesion to the vascular wall and platelet aggregation by activation of cGMP/PKG pathway, which in turn leads to reduction in concentration of Ca^2+^ with vasodilation and increased blood flow.^8^, ^9^ Nonetheless, a common feature of endothelium dysfunction is reduced NO bioavailability, which induces abnormal platelet activation leading to thrombus formation and vessel occlusion.

**Figure 1:**
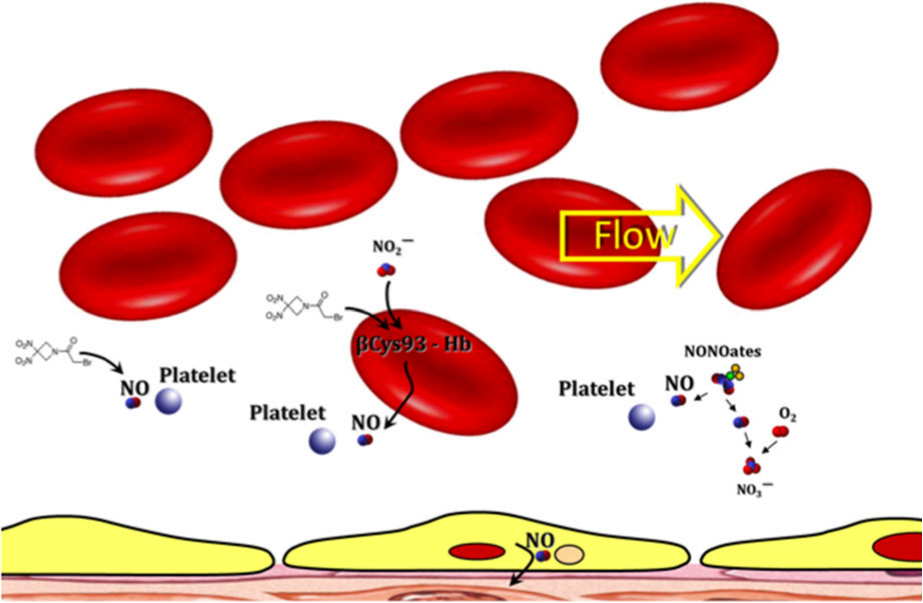
A Diagram, which Depicts Four Methods of NO Release, Leading to Increased Blood Flow: (a) nitric oxide is postulated to be released from RRx‐001 (formula shown below) directly via a Nef‐like reaction; (b) RRx‐001 binds to the betaCys93 residue on haemoglobin, which accelerates the formation of nitric oxide from deoxyhemoglobin in RBCs; (c) spontaneous release of nitric oxide from a NONOate in aqueous media; (d) production of NO via eNOS in the endothelium

RRx‐001, a new class of aerospace‐derived anticancer and normal tissue cytoprotective agent,^10^ binds selectively and irreversibly to an accessible and highly conserved thiol on haemoglobin called beta (β) cysteine 93; this binding of RRx‐001 to Hb β cysteine 93 significantly accelerates the rate of reduction in endogenous nitrite, which RBCs carry in bulk,^11^ to nitric oxide.^12^, ^13^ In addition, to a much lesser extent, one of the nitro groups on RRx‐001 is postulated to be the site of a Nef‐like reaction, a standard reaction of organic nitro derivatives,^14^ wherein nitric oxide is non‐enzymatically released. This RRx‐001‐mediated hyperinduction of NO^15^ under hypoxic conditions and lack of hypotensive side‐effects as well as headache, facial flushing, etc.^16^ differentiates it from other nitric oxide donors such as organic nitrates, S‐nitrosothiols, diazeniumdiolates‐NONOates, furoxans, zeolites, NO hybrid drugs and hydroxyurea^17^ that act systemically, resulting in potential toxicities.^18^ The primary objective of this study was to investigate the potential antiaggregant and antithrombotic effects of RRx‐001, as an NO donor, and to compare these ex vivo effects in whole blood and platelet‐rich plasma (PRP) with those of diethylenetriamine NONOate (DETA‐NONOate), which spontaneously releases nitric oxide in aqueous solution. A diagram, which depicts NO release from RRx‐001, DETA‐NONOate and eNOS in the endothelium, is shown in Figure 1.

## 2 METHODS

### 2.1 Blood and reagents

Blood was collected from healthy volunteers after fasting state of at least 3 hours. The venous blood samples were taken from healthy volunteers, of 24 (mean 3 SD) years of age in tubes containing sodium citrate in the ratio of 9:1 (vol per vol). Sodium nitrite (NaNO_2_), adenosine 5′diphosphate (ADP), collagen and Dextran 500 were purchased from Sigma (St Louis, MO, USA). DETA‐NONOate (diethylenetriamine NONOate) was purchased from Cayman Chemical (Ann Arbor, MI, USA). RRx‐001 was obtained from EpicentRx (San Diego, CA, USA). The nitric oxide inhibitor 2‐(4‐carboxyphenyl)‐4,4,5,5‐tetramethylimidazole‐1‐oxyl 3‐oxide (Carboxy‐PTIO) was purchased from Sigma (St Louis). Luciferin‐luciferase reagent (Chrono‐Lume) was purchased from Chrono‐Log Corporation (Howertown, PA, USA). DETA‐NONOate was freshly prepared by dissolving in 0.01 mol/L NaOH and used within 1 day. Immediately before use, DETA‐NONOate was diluted in phosphate‐buffered saline (PBS, pH 7.4). Sodium nitrite, ADP and Carboxy‐PTIO were prepared in PBS at pH 7.4. Collagen was prepared in deionized water. Monoclonal antibodies, FITC‐labelled anti‐human CD41a and PE‐labelled anti‐human CD62P, were purchased from Becton Dickinson (San Jose, CA, USA).

### 2.2 RBC aggregation

Red blood cells were separated from the blood by centrifugation at 2000 *g* for 7 minutes and washed 3 times with 10 mmol/L phosphate‐buffered saline (PBS) (pH = 7.4). The washed RBCs were then resuspended in PBS at a haematocrit of approximately 10%. RBC suspensions were divided into aliquots and exposed to either RRx‐001 (10 mg/kg), Dextran 500 (10 mg/kg) used as a positive control to induce RBC aggregation or PBS (control sample) at 25°C for 10 minutes. The degree of RBC aggregation was determined from duplicate measurements on a 20 μL blood sample with a photometric rheoscope (Myrenne Aggregometer^®^, Myrenne GmbH, Roetgen, Germany), which gives the indices of RBC aggregation “M” during stasis after shearing at 600 s^−1^. Briefly, this device measures the changes in light transmission, which are observed when sheared RBC suspensions are abruptly brought to a full stop. The decrease in the optical signal reflects the formation of RBC aggregates.

### 2.3 Coagulation studies

Coagulation studies were performed on plasma separated at 4°C from citrated whole blood.

Measurement of the activated prothrombin time (aPTT), prothrombin time (PTT), fibrinogen and platelets was determined by standard commercial assays. Platelet aggregation was analysed on an aggregometer. Platelets from PRP were incubated at 37°C, subjected to stirring and stimulated with agonists: 1 mmol/L ADP, 3 mmol/L ADP, collagen and epinephrine. The effect of vehicle (no treatment) and RRx‐001 (10 mg/mL) on the various tests was determined.

### 2.4 Platelet aggregation and ATP release

Whole blood was centrifuged at 1100 *g* for 10 minutes. The upper portion was collected for PRP. The lower portion was centrifuged further at 21 000 *g* for 10 minutes to obtain the platelet‐poor plasma (PPP). The PRP was used for aggregation studies within <3 hours after blood drawing. The effect of sodium nitrite, RRx‐001 and DETA‐NONOate on platelet aggregation in PRP and RP + erythrocytes was determined by the turbidimetric and impedance aggregometry, respectively. In turbidimetric experiments, the PRP was pre‐incubated with 0.01‐100 mmol/L sodium nitrite, RRx‐001 or DETA‐NONOate at 37°C for 5 minutes. In separate experiments, the PRP was pre‐incubated with 0.01‐100 mmol/L sodium nitrite, RRx‐001 or DETA‐NONOate at 37°C for 5 minutes in the presence 200 mmol/L Carboxy‐PTIO. Platelet aggregation was induced by 8 mmol/L ADP. Platelet aggregation was measured using a Platelet Aggregometer Model 560 (Chrono‐Log Corporation). The increased light transmission was an indicator of increased aggregation in PRP. In the impedance experiments, platelet aggregation in PRP or PRP + erythrocytes was studied by impedance aggregometry in which the change in electrical impedance (in ohms, Ω) was monitored. The increase in the electrical impedance of electrode correlated with increased aggregation. The erythrocytes were added into PRP to produce a haematocrit of 1% and 20%. The haematocrit values in the mixture of PRP + erythrocytes were confirmed by the microhaematocrit centrifugation. The PRP+erythrocyte samples were pre‐incubated at 37°C with 0.01‐100 mmol/L sodium nitrite, RRx‐001 or DETA‐NONOate at 37°C for 5 minutes in the presence or absence of Carboxy‐PTIO. The ATP released from platelets was monitored by luminescence aggregometer using a luciferin‐luciferase assay. The amount of ATP was calculated from the luminescence signal of ATP standard according to the manufacturing protocol. Additional, platelet aggregation with 0.01‐100 mmol/L sodium nitrite using impedance measurements, in PRP or PRP + erythrocytes, was performed at haematocrit of 20% of pre‐incubated samples with RRx‐001 or DETA‐NONOate at 37°C for 5 minutes in the presence or absence of Carboxy‐PTIO. Lastly, platelet aggregation with 0.01‐100 mmol/L sodium nitrite during normoxia (room air) and hypoxia (5% O_2_) using impedance measurements, in PRP or PRP + erythrocytes were performed at haematocrit of 20% of pre‐incubated samples with RRx‐001 or DETA‐NONOate at 37°C for 5 minutes in the presence or absence of Carboxy‐PTIO.

### 2.5 Measurement of cGMP levels in platelets

Washed platelets were prepared from PRP and resuspended in the incubation buffer (130 mmol/L NaCl, 12 mmol/L NaHCO_3_, 0.34 mmol/L NaH_2_PO_4_, 2.9 mmol/L KCl, 1 mmol/L CaCl_2_, 0.8 mmol/L MgCl_2_, 5 mmol/L Hepes, 5 mmol/L glucose, pH 7.4). The washed platelet number was adjusted to 4500 cells/mL and incubated for 1 hour at room temperature before use. 0.1 mmol/L nitrite was added into the platelet suspension in the presence or absence of erythrocytes (20% haematocrit), and in the presence or absence of erythrocytes (20% haematocrit) pre‐incubated with RRx‐001 for 5 minutes. After incubation with agonist and stirring at 37°C for 5 minutes, the samples were mixed with 5% trichloroacetic acid and centrifuged at 18 000 *g* for 10 minutes. The supernatant was collected and stored at 80°C until measurement. The cGMP levels were measured by the enzyme‐linked immunoassay (Cayman Chemical, Ann Arbor, MI, USA).

### 2.6 Nitrite/nitrate levels in samples

Plasma proteins were removed by adding an equivolume of methanol and centrifuged at 5: 15000 RPM (0.025 Gs) at 4°C. Concentrations of nitrite and nitrate in the supernatant were measured with a NOx analyser (ENO‐20; Eicom, Kyoto, Japan). This analyser combines the Griess method and high‐performance liquid chromatography.

### 2.7 Statistical analysis

All data are means ± SD. Statistical analysis was analysed by GraphPad Prism version 6 (GraphPad software Inc., San Diego, CA, USA). Correlation was analysed by Pearson's method. ANOVA with Tukey's multiple comparison were used to compare with acceptable *P*‐value, 0.05.

## 3 RESULTS

### 3.1 RRx‐001 had no effect on RBC rouleaux formation or aggregation

**Figure 2:**
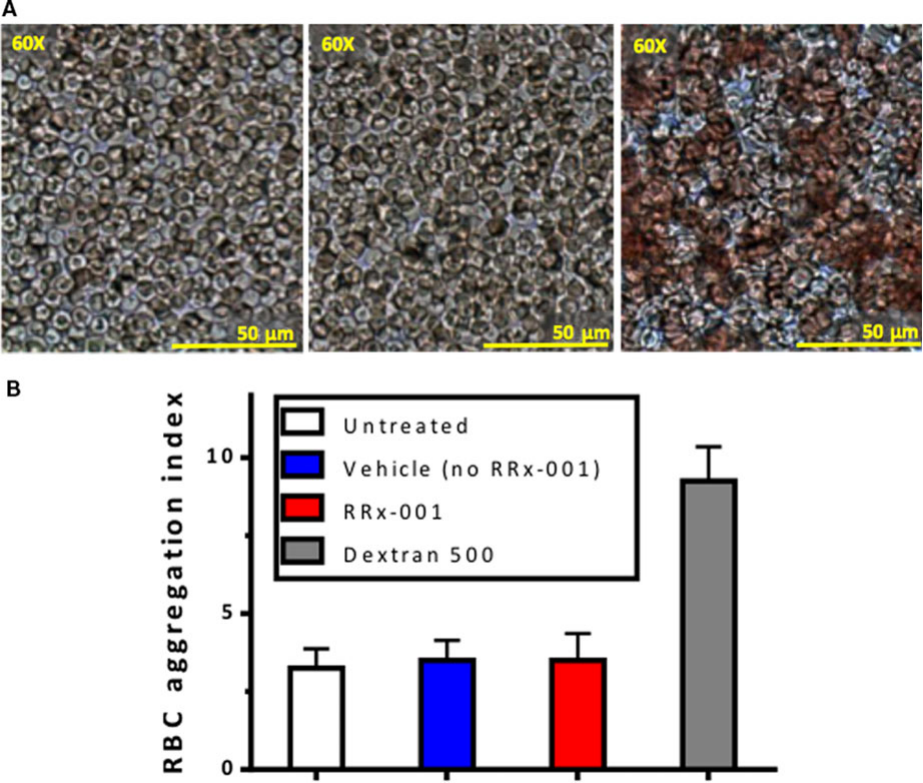
A, Blood Smear for Aggregation Studies Obtained from Heparinized Human Blood Untreated or Treated with Vehicle, RRx‐001 (10 mg/kg) or Dextran 500 kDa (10 mg/kg in Sterile Saline, Positive Control). From left to right vehicle‐treated blood, RRx‐001‐treated blood and Dextran‐treated blood. Clumping and rouleaux formation is only seen with Dextran, the positive control; B, RBC aggregation index of human blood untreated, or treated with Vehicle, RRx‐001 (10 mg/kg) or Dextran 500 kDa (10 mg/kg in sterile saline, positive control). RBC aggregation index was unchanged between untreated blood, vehicle‐treated blood and RRx‐001 treated blood. Only Dextran, the positive control, showed a significant (*P* < 0.05) increase in RBC clumping and aggregation (far right panel)

In red blood cells exposed to RRx‐001, no rouleaux formation or increased aggregation was observed compared with vehicle or untreated RBCs, as shown in Figure 2.

### 3.2 RRx‐001 modified ADP, collagen and epinephrine‐induced platelet aggregation but not coagulation parameters

**Figure 3:**
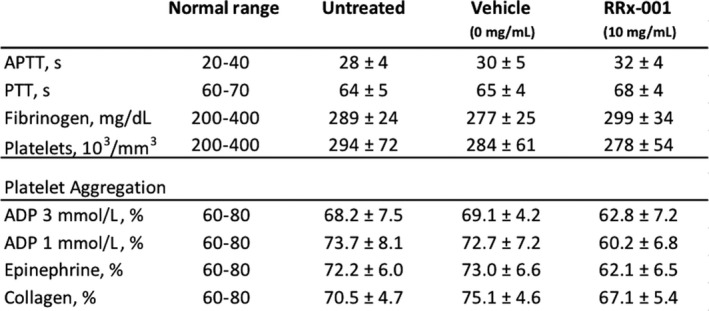
Basic Coagulation and Platelet Aggregation Parameters with Untreated, Vehicle‐treated and RRx‐001‐treated Blood. RRx‐001 does not Affect APTT, PTT, Fibrinogen or Platelet Numbers Compared to Untreated Blood or Control. However, RRx‐001 appeared to attenuate platelet aggregation in response to ADP, collagen and epinephrine. ADP, adenosine diphosphate; APTT, activated partial thromboplastin time; PTT, partial thromboplastin time

At concentrations of 10 mg/mL, RRx‐001 did not affect coagulation parameters (APTT, PTT and fibrinogen were all within normal range) and, similarly, thrombocyte count was within the normal range (278 ± 54 × 10^3^/mm^3^). However, RRx‐001 non‐significantly attenuated or modified platelet adhesion/aggregation induced by collagen, epinephrine or ADP, as shown in Figure 3.

### 3.3 The platelet inhibitory effects of RRx‐001 and DETA‐NONOate are reversed by the addition of CPTIO, a nitric oxide scavenger

**Figure 4:**
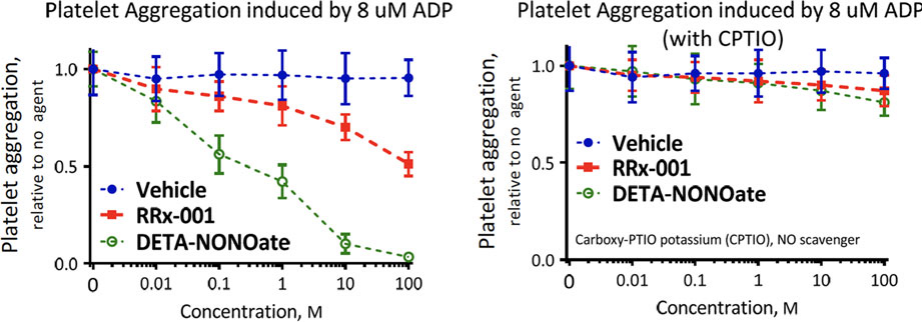
Platelet Aggregation of Platelet‐rich Plasma Measured using % of Light Transition. Effects of RRx‐001 and DETA‐NONO in the absence (left panel) and in the presence (right panel) of the nitric oxide scavenger, CPTIO. Each point represents the mean ± SEM of all experiments. The platelets were incubated with test drugs for before stimulation with ADP, and aggregation was monitored thereafter. Results are expressed as percent inhibition of aggregation relative to the untreated platelets CPTIO: 5,2‐(4‐carboxyphenyl)‐4,4,5,5‐tetramethylimidazoline 1‐oxyl 3‐oxide

Platelet aggregation was studied in platelet‐rich plasma (PRP). In PRP, DETA‐NONOate and RRx‐001 both exhibited a dose‐dependent inhibition of platelet aggregation induced by 8 μmol/L ADP; however, the effect of DETA‐NONOate on platelet aggregation was much more pronounced that RRx‐001. However, the antiplatelet effects of both DETA‐NONOate and RRx‐001 were reversed by CPTIO, an NO scavenger, which suggests an NO‐mediated action. (Figure 4).

### 3.4 The platelet inhibitory effects of RRx‐001 and DETA‐NONOate are dependent on haematocrit

**Figure 5:**
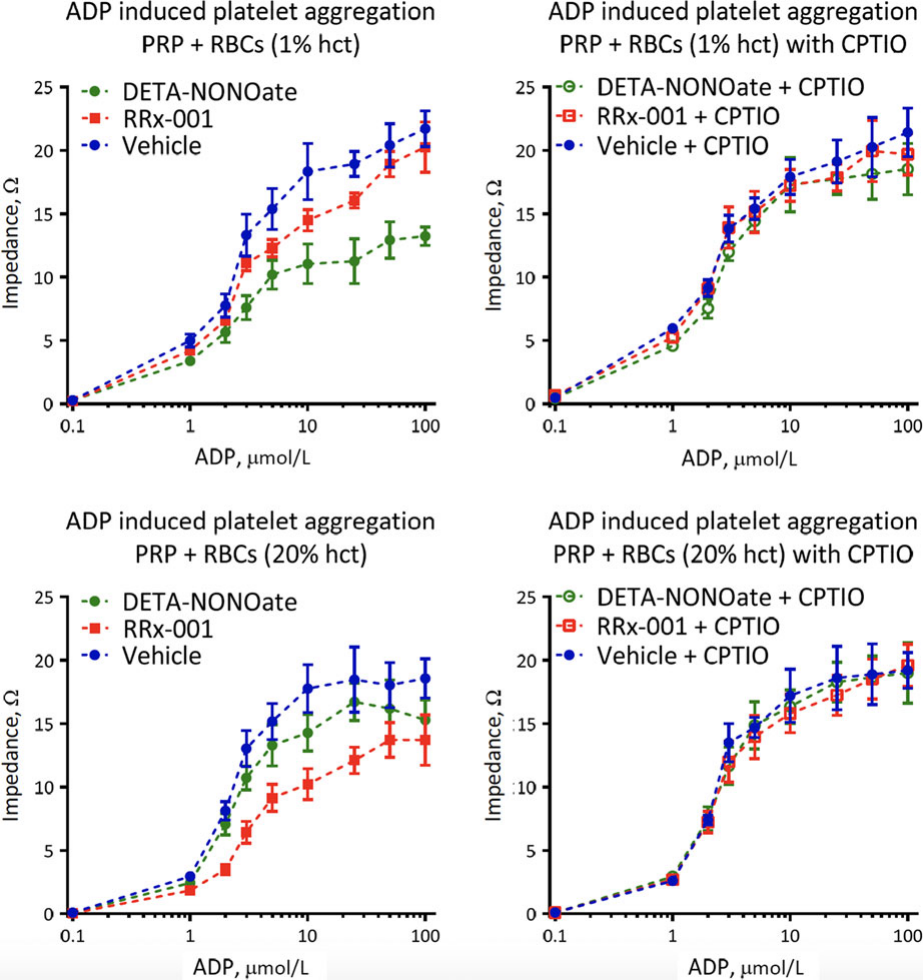
Inhibition of Platelet Aggregation by DETA‐NONO Decreased in the Presence of Increasing Haematocrit whereas Inhibition of Platelet Aggregation by RRx‐001 Increased in the Presence of Increasing Haematocrit. Platelet aggregation was induced by 8 μmol/L ADP. CPTIO completely reversed the inhibition. All experiments were performed at 37°C

In platelet aggregation measured by impedance (electrical resistance), the magnitude of effect for RRx‐001 and DETA‐NONOate varied inversely with each other depending on the haematocrit. As RRx‐001 stimulates red blood cells to reduce nitrite to NO and since haemoglobin scavenges nitric oxide, the effect of haematocrit on platelet inhibition was tested. The impedance aggregometry method was used because RBCs interfered with the light transmission method. The inhibitory effect of DETA‐NONOate on platelet aggregation decreased in the presence of RBCs with a 20% haematocrit attenuating the effect of DETA‐NONOate to a greater extent than a 1% haematocrit, presumably due to NO destruction by haemoglobin in RBCs. By contrast, the inhibitory effect of RRx‐001 on platelet aggregation increased in the presence of RBCs with a 20% haematocrit > than 1% haematocrit, likely due to stimulation of the nitrite reductase function of deoxyhaemoglobin, which converts nitrite to NO. However, irrespective of the haematocrits, the antiplatelet effects of both DETA‐NONOate and RRx‐001 were reversed by CPTIO. (Figure 5).

### 3.5 The addition of nitrite enhanced the inhibitory action of RRx‐001 on platelet activation

**Figure 6:**
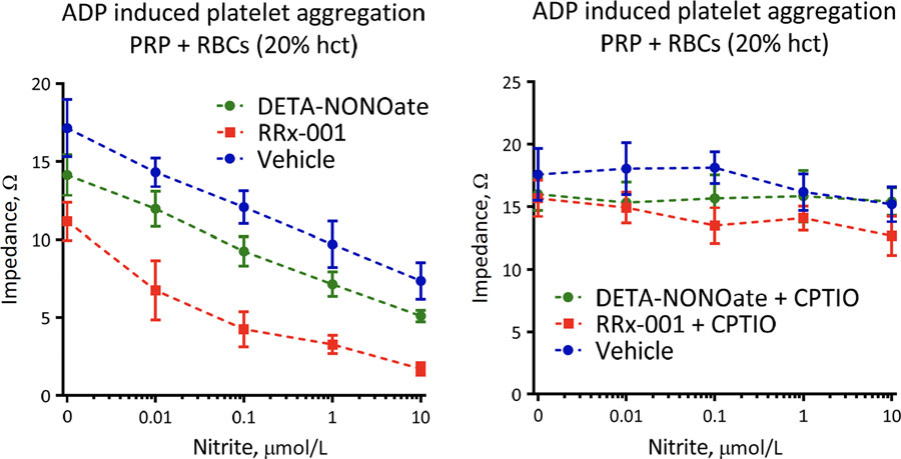
Nitrite+ red blood cells (RBCs) (20% Haematocrit) Inhibited ADP‐induced Platelet Aggregation. Nitrite was incubated in PRP + RBCs in the presence or absence of CPTIO before induction of aggregation by ADP

The potential effect of nitrite addition (0.1‐10 μmol/L) on the RRx‐001‐mediated regulation of platelet activity, given that RRx‐001‐bound deoxyhaemoglobin reduces nitrite to NO, was examined. In the presence of RBCs (20% haematocrit), increasing concentrations of nitrite inhibited platelet aggregation for all 3 groups: vehicle, RRx‐001 and DETA‐NONOate. However, the effect of nitrite on RRx‐001‐inhibition was significantly greater compared to DETA‐NONOate. In addition, the effect of nitrite on all 3 groups was diminished in the presence of CPTIO (Figure 6).

## 4 DISCUSSION

The main objective of this pilot study was to investigate ex vivo the effects of RRx‐001 and DETA‐NONOate on platelet aggregation induced by ADP, epinephrine and collagen in whole blood and PRP. The antiplatelet effects of RRx‐001 and DETA‐NONOate varied depending on the dose and presence or absence of RBCs. Consistent with its red blood cell‐based mechanism of action^19^ in which RRx‐001 binds to Hb and catalyses the conversion of nitrite to NO^.^ from deoxyhaemoglobin, the antiplatelet effect of RRx‐001 increased at a higher haematocrit and was further enhanced with the addition of nitrite; however, no effect on coagulation parameters or RBC aggregation was observed. These doses of RRx‐001 correspond to its plasma concentrations in patients with cancer.

By contrast, the antiplatelet effect of DETA‐NONOate was more pronounced in pure PRP and decreased as the haematocrit increased since haemoglobin‐containing RBCs scavenge nitric oxide and reduce its availability. The antiaggregatory actions of both RRx‐001 and DETA‐NONOate were almost completely abrogated in the presence of the NO inhibitor, CPTIO, irrespective of whether RBCs and nitrite were present, which served to confirm the NO‐dependent mechanism of action.

These antiaggregatory properties of RRx‐001, as an antitumour agent in Phase 3 clinical trials, merit in vivo correlation, especially as platelet activation is known to contribute to tumour invasion and metastasis^20^ and a number of anticoagulants and antiaggregatory agents have already been investigated for their possible anticancer effects. While these ex vivo platelet aggregation studies are at best a crude indicator of in vivo activity and involved blood collected from healthy volunteers rather than hypercoagulable cancer patients, it is nevertheless possible to interpret the results in the context of multiple clinical trials for several different tumours types including small cell lung cancer (SCLC), non‐small cell lung cancer (NSCLC), glioblastoma (GBM), neuroendocrine and ovarian cancer in which so far iegoonly one patient of 170 that has received RRx‐001 developed an arterial thromboembolic event (cerebrovascular accident or CVA), resulting from enhanced platelet aggregation. From these experiments, it seems reasonable to conclude that any relationship between RRx‐001 and the CVA event in this one patient is improbable (although not impossible).

Currently, RRx‐001 is dosed as a single agent and in combination with standard chemotherapy and radiation; its mechanism of anticancer action is thought to be related to pan‐epigenetic inhibition^21^, ^22^ and the repolarization of tumour associated macrophages and neutrophils^23^; however, the synergistic activity between nitrite and RRx‐001 on platelet aggregation suggests that co‐administration with nitrite, which would lead to overproduction of NO^.^ and other genotoxic reactive nitrogen species under the hypoxic conditions presumably endemic only to tumours, may not only result in enhanced cytotoxicity but also improved safety, as patients with cancer are prone to increased platelet activation and thromboembolic events.

In conclusion, these data, which confirm previous observations that RRx‐001 catalyses the conversion of nitrite to nitric oxide from deoxyhaemoglobin^24^ suggest that RRx‐001, apart from its anticancer effects and unlike all other known NO‐donating agents, reduces platelet aggregation and protects against thromboembolism as haemoglobin and haematocrit levels increase. Moreover, also unlike other known NO‐donating agents, because RRx‐001 mediates increased nitric oxide production under hypoxic conditions, NO is released locally on demand where it is most needed when eNOS is impaired.

## CONFLICT OF INTEREST

The authors disclose that EpicentRx, Inc. funds research of RRx‐001.
